# Public discourse narratives: from ‘Secret Aid Worker’ discontent to shifting power in humanitarian systems

**DOI:** 10.1111/disa.12651

**Published:** 2024-07-15

**Authors:** Hannah Strohmeier, Unni Karunakara, Catherine Panter‐Brick

**Affiliations:** ^1^ Institute of International Health Charité – Universitätsmedizin Berlin Germany; ^2^ Global Health Justice Partnership Yale Law School United States; ^3^ Yale School of Public Health United States; ^4^ Jackson School of Global Affairs Yale University United States; ^5^ Department of Anthropology Yale University United States

**Keywords:** aid workers, coloniality, discourse, duty of care, power, racism, social change

## Abstract

Public discourse is rich in meaning, reflecting consensus, dissent, and change. Yet, very little public discourse on the humanitarian sector has been authored by aid workers themselves. We conducted a thematic analysis of the ‘Secret Aid Worker’ (SAW) series, published in *The Guardian* newspaper between 2015 and 2018, the only corpus of data on humanitarian life experiences publicly accessible through mainstream media. Our research questions were twofold: how did authors frame their work and appraise humanitarian structures?; and how did they reflect and amplify humanitarian issues of the time? The main themes included: personal challenges of humanitarian life; characterisation of stakeholders; and systemic issues within the humanitarian sector. The SAW narratives reveal a powerful discourse of discontent. They planted seeds of change regarding shifting power, coloniality and racism, sexual abuse, and duty of care. We argue that such public discourse has symbolic power, calling for greater accountability, equity, and justice in remaking the future of the humanitarian sector.

## 1 INTRODUCTION

Aid workers are tasked with the delivery of critical assistance in humanitarian settings. This requires diverse managerial and technical expertise, in the form of project accounting, advocacy, communications, human protection, and security management. Globally, aid workers come from a range of backgrounds, have diverse motivations, and numerous career goals, and are variously stationed in crisis environments and organisational headquarters. This makes the framing of humanitarian aid workers, as a group, a challenging task (Bortolotti, 2004; Fechter and Hindman, 2011; Gritti, 2015; Strohmeier, 2019). Historically, they have been depicted as selfless people, acting because of human compassion, oftentimes living a life of extremes (Malkki, 2015). Especially in the Global North, lay representations of humanitarian workers go hand in hand with altruistic motivations, heroic traits, and international engagement (Bortolotti, 2004; Kleinman, 2007; Alexander, 2013; Malkki, 2015). In the words of Houldey (2017, p. 1), ‘the common idea of the aid worker is of a selfless soul who travels far from home to an unfamiliar and challenging environment, giving up a more privileged existence in their own country’.

This portrayal of international aid workers has been vigorously critiqued with respect to neoliberal and neocolonial assumptions about human needs, power, solidarity, and vulnerability. Noble (2013, p. 3), for instance, highlighted images conveying that ‘the powerful liberal heroes are saving the helpless, weak victims’. The notion of beneficence, motivated by human compassion and construed as humanitarian solidarity and a moral response to suffering and injustice, has been especially strongly contested (Fassin, 2007; Bornstein and Redfield, 2011; Ticktin, 2016; Chouliaraki, 2019). Over the past decade, social scientists have called for a closer empirical and theoretical examination of the core principles, underlying behaviours, and forms of intervention characterising humanitarian action (see, for example, Kleinman, 2007; Mosse, 2011; Redfield, 2013; Abramowitz and Panter‐Brick, 2015; Allen, 2015; Malkki, 2015; Roth, 2015). More specifically, they have investigated the interactions between first‐hand narratives and everyday practices, to explicate what these can reveal about local moral worlds (Kleinman, 2007), the subjectivities of humanitarian workers (Good et al., 2014), ethics and governance (Abramowitz, Marten, and Panter‐Brick, 2014), and forms of power (James, 2022).

While scholarly analyses of humanitarianism abound, very little public discourse on the inner workings of the humanitarian sector has been authored by aid workers themselves. Cain, Postlewait, and Thomas (2004), Orbinski (2008), and Alexander (2013) are among the very few humanitarian workers who have published autobiographical books about their work‐life experiences (in English). For her part, Bergman (2003) edited a much‐cited anthology focusing on humanitarian life experiences, consisting of short narratives by international and local aid workers reflecting on the extraordinary nature of ordinary humanitarian work. A small number of blogs, written by aid workers on publicly accessible online media platforms such as AidSpeak, were launched, but have now ceased. The podcasts and newsletters published by The New Humanitarian, a non‐profit newsroom founded by the United Nations (UN) in 1995 to provide on‐the‐ground reporting of humanitarian crises, have revived a form of commentary and network‐led communication among scholars and practitioners. Despite these means of communication, though, few aid workers have documented their lived experiences in widely available, public discourse formats.

Our study analyses the corpus of public discourse narratives produced by humanitarian workers between 2015 and 2018 and published by *The Guardian* under the title of the ‘Secret Aid Worker’ (SAW) series. *The Guardian* is one of Great Britain's leading newspapers, ‘globally renowned for its coverage of politics, the environment, science, social justice, sport and culture’ (*The Guardian*, n.d.). The series was published as part of its global development category forum, funded by the Bill & Melinda Gates Foundation. It comprises a collection of short narratives, on topics chosen by individual aid workers themselves. The posts were neither curated nor vetted by humanitarian employers. They are a popular read among aid workers, and a point of reference for scholarly research (see, for example, Scarnecchia et al., 2017; Holden et al., 2018–19; Strohmeier, Scholte, and Ager, 2018; Strohmeier and Panter‐Brick, 2020; Cooper, 2021). This is the only pool of data on humanitarian life experiences that is publicly accessible, in an anonymised format, and available through a mainstream medium. As such, it constitutes a unique historical archive of humanitarian culture, revealing how humanitarians view their roles and their sector.

### 1.1 Issues of the time

The period during which the SAW articles were published was marked by several historically important humanitarian, social, and political developments. We draw attention to three main issues which both impacted aid workers and put the spotlight on ethical and systemic issues within the humanitarian sector (see Table 1). The first issue is a failure in duty of care: organisational responsibility for the safety of staff and for measures taken to mitigate harm and enhance security. In 2015, the Norwegian Refugee Council (NRC) was sued by Steven Patrick Dennis, a Canadian aid worker who had been kidnapped in the Dadaab refugee camp, Kenya. The courts ruled that the NRC was responsible for gross negligence and liable for compensation, a verdict that was recognised as a ‘wake‐up call’ for the industry (Young, 2015). Furthermore, during the 2014–16 Ebola outbreak in West Africa, the World Health Organization (WHO, 2015) reported that health workers were 21–32 times more likely to be infected with the virus than adults in the general population. The critical need to protect health workers in fragile health systems underlined the obligation of organisations to protect staff from unreasonable harm (McDiarmid and Crestani, 2020).

**TABLE 1:** Three main issues marking discourses in humanitarian public media between 2015 and 2018.

Issue 1: failure of duty of care	Issue 2: inequitable operation of power	Issue 3: condoning of abuse
In 2012, Steven Patrick Dennis, a Canadian humanitarian worker, was kidnapped and taken into Somalia while working for the Norwegian Refugee Council (NRC) in Dadaab refugee camp. He experienced physical injuries and severe mental illness as a consequence (Dennis, 2015). In 2015, Dennis filed two claims against NRC with the Oslo courts: one for support of losses; and one for gross negligence (Dennis, 2015). Later that year, Dennis won the case, which ‘has been hailed a landmark ruling and wake‐up call for the industry’. The verdict ‘found the NRC liable for physical and psychological injuries and awarded compensation for gross negligence’ (Young, 2015). His story ‘has become known around the world, especially in the humanitarian sector’, as it set ‘an unexpected precedent for aid workers’ (Riglietti, 2023). Also in 2015, WHO reported a disproportionally high risk of health workers contracting Ebola, underlining the organisation's obligation to protect staff from harm (WHO, 2015). Since then: ‘It is undeniable that [humanitarian] organizations of all sizes have revisited their security structures’ (Clamp, 2022).	At the 2016 World Humanitarian Summit (WHS), thinking emerged that humanitarian responses should be ‘as local as possible, as international as necessary’. Numerous changes were proposed at the WHS concerning how donors and organisations can achieve localisation. These are known as the ‘Grand Bargain’ (OCHA, n.d.). The Grand Bargain has since been described as ‘a unique agreement between some of the largest donors and humanitarian organisations who have committed to improve the effectiveness and efficiency of the humanitarian action, in order to get more means into the hands of people in need’ (IASC, n.d.). The discourses of anti‐racism and decolonisation and anti‐racism at times overlap with localisation discourse (Robillard, Atim, and Maxwell, 2021). Since then: calls for decolonisation and anti‐racism have loomed large in humanitarian public fora; such discourses were amplified in 2020 by the deaths of George Floyd and other Black and brown people in the United States and the re‐emergence of the Black Lives Matter (BLM) movement (see, for example, Aloudat, 2021; Khan, 2021; Khan, Dickson, and Sondarjee, 2023).	In 2018, a series of allegations of misconduct were levelled at staff of various aid organisations, including OXFAM and Save the Children—often referred to as ‘sex scandals’ in the media. These scandals ‘brought unprecedented attention to the issue of sex abuse in aid’ (Naik, 2022, p. 21). Shortly afterwards, also in 2018, humanitarian workers started the #AidToo movement as ‘a response to the prevalence of sexual harassment, abuse, and exploitation within the aid industry’ (Sauter, 2024). #AidToo drew inspiration from #MeToo, a movement that grew to prominence after actress Alyssa Milano posted the hashtag on Twitter in 2016, to encourage women to share their experiences of assault and abuse (Riley, 2020). Since then: many organisations have focused on safeguarding and have invested large amounts of money in research, training, and the improvement of internal oversight, investigation, and reporting mechanisms (Naik, 2022).

**Source:** authors.

The second issue is the inequitable operation of power by states and humanitarian actors, resulting in widespread concern regarding hierarchies of power, colonial practices, and social exclusion of local actors from agenda‐setting. In 2016, the World Humanitarian Summit (WHS) adopted localisation as a key commitment, to address the historic exclusion and marginalisation of local humanitarian actors in decision‐making. Institutionally, white privilege and the inequities baked into human resource policies resulted in locally‐ or nationally‐hired staff consistently being excluded from decision‐making and career advancement. Common reforms sought to include fostering more equitable partnerships, increasing access to funding, and promoting local humanitarian leadership (Robillard, Atim, and Maxwell, 2021).

The third issue, the condoning of abuse, especially sexual abuse, created an environment that harmed staff and lacked accountability. In 2018, several aid organisations, including Oxfam and Save the Children, faced allegations of misconduct, depicted in the media as ‘sex scandals’. These incidents drew unprecedented attention to the issue of sexual abuse within the aid sector, prompting humanitarian workers to launch the #AidToo movement to address sexual harassment, abuse, and exploitation within humanitarian organisations (Naik, 2022).

### 1.2 Secret Aid Worker discontent

In this paper, we identify contested topics that humanitarian workers chose to reflect upon publicly, in the SAW series, at the time. We argue that the SAW series represents a form of storytelling to a largely lay audience, conveying dark secrets about the challenges of humanitarian life and oppressive humanitarian structures. Anonymously published writing manifestly allowed space for recrimination and reproach, as well as for frank appraisal of shortcomings in the humanitarian sector. The articles were rare public expressions of discontent from within the aid sector, and provided an impetus for change through soul‐searching, the airing of dirty laundry, and anonymous public communication. Their discourse had symbolic power, calling for greater accountability, equity, and reform in the humanitarian sector.

Analysis of the SAW narratives, in the light of contemporaneous discourses in humanitarian public media, allows us to understand how humanitarians construct their roles and comprehend the structures of humanitarian engagement. It also permits us to highlight the value of public discourse in bringing about change in the humanitarian sector. Specifically, our paper presents a thematic analysis of all 95 SAW submissions, and then discusses findings vis‐à‐vis recent online writing in the humanitarian sector, placing specific emphasis on change and continuities. Our research questions were twofold:

1. How did authors frame their work and appraise humanitarian structures, in writing the ‘Secret Aid Worker’ narratives?
2. How did this public discourse reflect and amplify contentious humanitarian issues of the time?

In the results section, we present three main themes pertaining to the corpus of data published in the SAW series. In the discussion section, we turn to efforts to reform and shift power within humanitarian systems, examining donor power, coloniality and racism, sexual abuse, and duty of care in humanitarian engagement. We assess how these issues were reflected in the SAW narratives and how they have been amplified since then. In doing so, we implement a rigorous social sciences approach, which, in the words of Schmidt (2011, p. 107), will ‘take ideas and discourse seriously’ and therefore help to ‘explain the dynamics of change (and continuity)’.

Our analysis is timely, given calls for a ‘rethink’ of models of humanitarian assistance to put actual ‘people’ squarely at the centre of humanitarian action (Bennett, 2018), and to emphasise a more relational, and less hierarchical, architecture of humanitarian assistance (Panter‐Brick, 2023). As argued by Bollettino et al. (2023, p. 1), ‘[a]id workers offer important perspectives for understanding better the most pervasive challenges that arise when implementing emergency programming in humanitarian settings’. These authors presented a thematic analysis of 4,679 applications of mid‐career aid workers to the National NGO Program on Humanitarian Leadership, over 13 application cycles between 2016 and 2022. The application included the question ‘what do you consider to be the biggest challenges in the implementation of emergency response programming in today's humanitarian settings?’, based on lived experiences in the humanitarian sector. Through qualitative coding of applicant responses, Bollettino et al. (2023) identified 14 major challenges, and then examined associations between reported challenges and respondent characteristics, using demographic data available from the application pool. This novel analysis provided a relative ranking of perceived barriers to the effective implementation of humanitarian response and indicated how major concerns—coordination, funding and human resources, security, localisation, and bureaucracy—varied by region of origin, type of aid organisation, and years of experience.

Still, very little public discourse, expressed by aid workers themselves, focuses on personal challenges, career expectations, and experiences of organisational structures. The 129 postings of *Médecins Sans Frontières (MSF) staff*, published *on the official United Kingdom website of the health‐focused international humanitarian organisation*, are one exception; they were written as ‘Letters from the Field’ from May 2002 to April 2012. Such postings were qualitatively analysed by Ager and Iacovou (2014) to identify recurrent themes within organisationally‐condoned, public narratives about humanitarian practice. These authors noted that ‘little systematic research has focused upon workers’ constructions of humanitarian practice’ (Ager and Iacovou, 2014, p. 431). Findings suggested that aid workers have motivations, norms, and expectations that include ‘making a significant social contribution, seeking more satisfying work, and searching for new experiences'; postings reflected a ‘negotiated script of personal and organizational understandings' (Ager and Iacovou, 2014, p. 438) of the humanitarian sector.

A granular reading of public discourse, written by aid workers, can thus reveal a great deal about the cultural, historical, and moral landscape in which humanitarian work is undertaken. Indeed, public discourse gives a sense of intergroup communication that allows for reflection and the reinforcement of opinions, attitudes, values, and behaviours in society (Marlow, 2017). Notwithstanding its inherent value, public discourse on the humanitarian sector, authored by humanitarian practitioners themselves, is rare.

## 2 METHODS

The SAW series includes 95 articles published by *The Guardian* between 2015 and 2018 (see Table S1 in the supplementary materials).^1^ The text corpus is publicly available. Since the SAW articles were written anonymously, it is not possible to determine accurately whether the authors were international or national staff or vouch for their veracity, let alone infer many other sociodemographic characteristics. This represents an important limitation of the dataset. Nonetheless, it is clear that most published texts represent the words of international staff, working in diverse fields such as health, refugee assistance, fundraising, human rights, or communications, within crisis settings in Africa and Asia (see Table S1 in the supplementary materials).

We conducted a reflexive thematic analysis of these textual data. This type of analysis allows one to focus inductively on content, that is, codes and themes derived from the data with no preconceptions or use of a formal codebook (Braun and Clarke, 2012, p. 58). We allowed for semantic coding, which focuses on the explicit content of the data, as well as latent coding, aimed at revealing the underlying meaning of the data with elements of interpretation. Multiple codes could be assigned to the same portion of data, a process known as simultaneous coding (Good, 2023). We followed the six‐step analysis approach suggested by Braun and Clarke (2012). As the data are in the public domain, and no human subjects were involved, the research did not require institutional review for ethics approval. We used NVivo, Release 1.7.1, to facilitate the analysis of textual data.

Following this thematic analysis, two co‐authors conducted an online search of public humanitarian discourse in July 2023, screening for posts that spoke to abuse, coloniality, duty of care, gender, power, racism, and sexual abuse in the aid sector. We searched the websites of major humanitarian outlets, such as the Centre for Humanitarian Action (CHA), Devex, the Humanitarian Practice Network, openDemocracy, and The New Humanitarian, for recent work debating issues in the humanitarian sector. We reviewed all texts to evaluate the extent to which themes expressed in the SAW series had amplified or waned in current humanitarian discourse.

## 3 RESULTS

**FIGURE 1:**
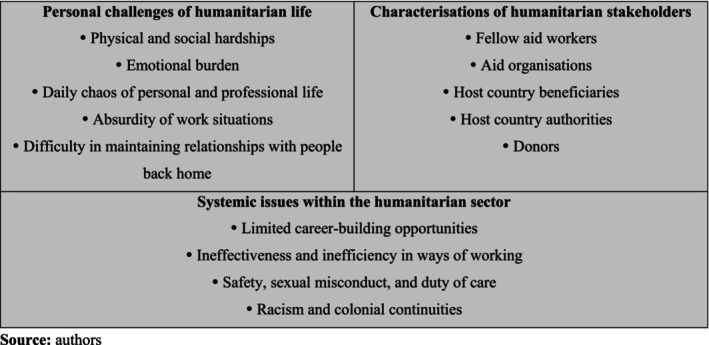
Thematic analysis of ‘Secret Aid Worker’ public data **Source:** authors

Our thematic analysis of SAW articles rendered three major themes (see Figure 1): (i) personal challenges of humanitarian life; (ii) characterisations of humanitarian stakeholders; and (iii) systemic issues within the humanitarian sector. We present these in turn, referring to verbatim data by their publication number in the SAW series.

### 3.1 Personal challenges of humanitarian life

Within this theme, narratives commonly contained ingenuous descriptions of humanitarian work environments, concerning, inter alia: (i) physical and social hardships; (ii) the emotional burden; (iii) the daily chaos of personal and professional life; (iv) the absurdity of work situations; and (v) the difficulty in maintaining relationships with people back home. For example, one author of a SAW article (see Table S1 in the supplementary materials) was quick to describe hardship, isolation, and discomfort in the following way: ‘My first real field experience was in Nepal, in a village in the middle of nowhere, where I shared a flat with Nepalese colleagues’ (No. 40). Reporting about Afghanistan in 2009–10, another author commented: ‘Based in Kandahar, then probably the most dangerous place in the country. .. with only non‐alcoholic beer allowed and very little on offer in entertainment, we had to get creative when we did get free time’ (No. 53).

For many aid workers, confronting the depth of human suffering entailed dealing with a pronounced emotional burden. One author wrote: ‘I've been in one place where conditions were so bad that I had to make a huge effort not to burst into tears when walking down the street for my first few days there’ (No. 2). Indeed, a substantial proportion of the texts focused on mental and physical health, with many authors describing how their well‐being had been compromised as a direct or indirect consequence of working in aid. Some authors explicitly labelled the mental health problems experienced. For instance:

*Six months after I left [the organisation] I was experiencing random anger, anxiety, crying, and exhaustion* (No. 22).

*Finally, I was diagnosed with PTSD [post‐traumatic stress disorder] and malaria complications that had led to permanent injuries. I learned I would never bear a child. My life was shattered* (No. 21).

Descriptions of daily life, in the personal and professional spheres, highlighted how chaotic and frustrating this could be. Authors wrote about the transience of aid work, such as: ‘I lived the normal chaos aid workers know so well: of new places, new faces, new emergencies’ (No. 21). This situation made it difficult to sustain romantic relationships and build a family. Authors also elaborated on how the nature of emergency responses spilled over into the urgent pace of workloads, notably: ‘Aid workers continue to get up and work six or seven days a week to address the overwhelming humanitarian needs in South Sudan’ (No. 26). One author, writing about the risks of social media and connectivity, reflected on the humanitarian industry, and one's position within it, as follows:

*Humanitarianism has always had a very blurred line between personal and professional. When you're out on a posting for months at a time your colleagues often become friends, work/life balance is an abstract concept, and your role as ambassador for your country/NGO [non‐governmental organisation]/all NGOs never really goes away* (No. 34).

Narratives describing the absurdity of work‐life were quick to follow descriptions of daily hardships and work/life balance. For example, one author applied a heavy dose of cynicism when describing institutional ‘rest and recuperation’ (R&R) practices in ‘conflict zone number five’, depicting it in the form of a letter to family and friends back home, tinged with pejorative language otherising local people and local coworkers:

*Sorry I couldn't make it home for the third year in a row. My programme manager said they really needed to take R&R in Bali [Indonesia] for the ninth time this year, and they're sleeping with the only other programme officer in the base, so little ol’ me gets to run the joint … The locals don't really celebrate Christmas in the same way we do back home, but the local staff and I are going to have a little party. They've promised to bring a healthy goat and we'll make a sauce out of expired tomato paste and some green herbs that were left behind by an old staff member. I'm sure it'll taste great after a few slugs of the local moonshine. Did you know they make it using the very same sorghum we distribute on behalf of the World Food Programme? They're such a versatile people* (No. 74).

Another example of absurd work situations was recounted by an international aid worker, writing about the overzealous responses of international NGOs in the wake of Typhoon Haiyan (2013):

*Some of the most memorable development activities I learned about on the island were related to open defecation. In order to compel communities to change their behaviours, and stop pooing outdoors, certain NGOs have drawn upon the power of shame and disgust. Two pretty powerful emotions. They actually make community members go to locally known open defecation spots, get them to pick up poo and bring it to the group, where it is looked at and discussed. It's placed next to food to really drive the point home* (No. 29).

This narrative conveys a criticism of overt humanitarian beneficence leading to paternalistic approaches to ‘compel’ social change in local communities, as well as to the entrapment of international aid workers in absurd situations.

Lastly, aid workers highlighted difficulties in maintaining their relationships with family and friends. The lack of understanding among those left behind was perceived as hard to endure, and the praise aid workers receive for their work posed a challenge for many:

*When I tell people that I am a gender adviser, they ask what it means. My mum imagines me running across a war zone, scooping up women in my arms and dodging bombs while I carry them to safety. My brother thinks I advise people about gender reassignment. My friend thinks I work with ‘battered women’* (No. 77).

*I have been declared a saint. Not by Pope Francis or the Catholic Church, but by my grandmother's bridge club, a born‐again Christian I met in an airport, a teacher and my old hockey coach. For those of us who have chosen a career in development, this informal canonisation by friends, family and strangers alike is a familiar experience. We know that whenever a conversation turns to work, comments such as ‘Your family must be so proud’, ‘That must be so fulfilling’ or the ever loftier ‘You're a saint’ will follow* (No. 70).

Authors frequently doubted their choice to work in aid and critically reflected on their motivation, which had changed over time. Initially driven by compassion and the desire to help those less privileged, many authors expressed a new sense of humbleness regarding their roles and possible contributions to alleviate human suffering. For instance:

*You might wonder why I keep doing it. The answer is simple, because now it's personal. I've come to terms with the fact that my ability to affect world injustice is minimal. I know this. I know I have to look into their eyes and try to comfort them there and then, because once I walk out the door chances are little to nothing will change. Still, the least I can do is try* (No. 14).

These narratives reveal that a common bottom line, for an aid worker, is to ‘at least try’ amidst the frustration of lived experiences and the chaos of life in complex humanitarian crises. They also reveal, however, the burden of unrealistic expectations that aid workers carry as normative expectations of their roles and positionality. Humanitarian workers painted a picture of their roles which highlighted the challenges and transience of aid work, as well as the overbearing frameworks of assistance for local populations. Back home, they experienced a disconnect between their lived experience of working in conflict zones, punctuated by R&R practices, and the hard‐to‐endure praise from friends and family, who portray their work as ‘fulfilling’ and their motivations as those of ‘a saint’.

Some of the narratives explicitly addressed issues of human suffering and world injustices, as well as entrapment in humanitarian ways of life. Humanitarians made sure to describe their own struggles with physical and mental health yet know the pace of work to be urgent and chaotic. However, their narratives rarely situated personal challenges in the context of the challenges faced by the local populations for which they worked. Aid workers also indulged in a certain amount of exoticising of national staff and local communities, seemingly acting within a humanitarian bubble without experiencing more diverse, sustained social interactions.

This raises a whole host of concerns with respect to the nature of humanitarian engagement. What comes across, time and again, is the mismatch between expectations of humanitarian life and lived experiences, underlining the importance of pre‐departure briefings and training. Many narratives also indicated the critical importance of ongoing staff support for the management of stress, anticipating and preventing traumatic events and moral injuries, and robust post‐assignment care to deal with residual harm.

### 3.2 Characterisations of humanitarian stakeholders

The SAW narratives also focused on characterising (and caricaturing) the principal stakeholders within the humanitarian sector. We present, in turn, self‐expressed views about interactions with (i) fellow aid workers, (ii) aid organisations, (iii) host country beneficiaries of aid, (iv) host country authorities, and (v) donors.

With respect to fellow aid workers, authors put considerable effort into portraying the personality type of their colleagues in the sector. They described themselves vis‐a‐vis others along lines of gender, seniority, duty station (headquarters versus the field), and type of contract (international versus national). Most descriptions centred on negative character traits and habits. For example, a castigation of high‐level officials went as follows: ‘High‐level is a group of mostly old, mostly white, mostly men from privileged backgrounds’ (No. 56). Narratives were offered as warning shots to others, as in these three accounts of Western aid workers characterised as ‘lifers’ or ‘addicts’, acting out ‘hyper’ in a showy performance of drama which ‘locals’ could barely fathom:

*Aid workers tend to belong in two camps. The first is people who never want to leave. These come in all ages and love the forever‐young life, forever wandering around hot‐spots, setting up shop in rodent‐infested containers or compounds and living a commune life with multiple, interchanging partners. Many of these are not particularly interested in starting a family, or already have a family back home that manages just fine without them. They are referred to as ‘lifers’. The second group cannot wait to leave, and often grumble loudly about it. These people often sound like heroin addicts about to enter rehab, explaining to family and friends that this latest one‐year contract will be their ‘last hit’* (No. 27).

*You do occasionally meet someone, who's been working in this field for decades, who does seem immune to compassion. Who no longer seems to really care about the people they work with and for. People who are, let's be frank, somewhat addicted to horrific disasters* (No. 2).

*If I had to describe the western expat aid workers I've worked with in one word, it would have to be ‘hyper’. Most of the time they're running around the office – hardly ever on the ground in the communities they're meant to work in – checking, controlling, advising, shouting, trying to help, working late into the night. I've seen locals stare at them like they are a TV show on fast forward* (No. 40).

Indeed, one author likened Western aid workers to the crew of *The A‐Team*, a television show popular in the 1980s:

*In today's world of humanitarian assistance, aid workers in high‐profile locations, delivering life‐saving help to besieged Syrian towns or fighting Ebola in west Africa, can be likened to Hannibal and his motley crew* (No. 44).

Turning to aid organisations, authors were frank, even savage, in their depiction of poor management, lack of accountability, and ill‐treatment of NGO staff and aid recipients. Scathing words were written about NGO management and motivation:

*Many years down the line I stand by this: the increasing security threats that we as humanitarian workers face would never lead me to quit my job. But the management at my organisation just might* (No. 37).

*How important are the victims for whom the NGO purports to speak? I don't know. I'm sure, however, that even the organisation's most meaningful work is at least partly driven by the self‐promotional desires of a vanity NGO and its leadership* (No. 66).

*Worse though is the feeling that much of what I'm asked to do by my superiors is ethically questionable. In a previous job with another big aid organisation, I was asked to make up quotes to ‘enhance’ beneficiary case studies* (No. 55).

Authors turned to describing the local ‘beneficiaries’ of aid using accounts of helplessness and hopelessness, more so than empathy. In commenting about famine‐stricken communities, they disclosed a sense of numbness surfacing from humanitarian encounters, such as: ‘I've seen more malnourished people than I can remember. The bones jutting out of people with Aids [acquired immunodeficiency syndrome], children whose hair has changed colour due to a lack of nutrients’ (No. 2). From where they stood in the chain of humanitarian assistance, they tended to characterise local people as ‘poor victims’. And when they confronted suffering, ignorance, and corruption, some perceived the burden of the job as ‘too much’. In the words of one author:

*When I began supervising a project for the rehabilitation of malnourished children in the Democratic Republic of the Congo's capital Kinshasa, then Zaire, I was faced every day with the hopelessness of children brought in after failed attempts by traditional healers from the village to our health units. I watched these children, like flickering bulbs slowly extinguishing their light, gradually fade. I used to return home and cry, the weight of visions of these poor victims, victims to ignorance and corruption was too much* (No. 16).

Host countries too exert power and shape the environment of humanitarian action. Authorities were mentioned in the context of corruption, repression, and incompetence. One author, promoted to support the local government as an adviser, was witnessing ‘fraud and corruption on a regular basis’ (No. 59). Another pointed to the fact that local ‘authorities would deny the extent of the crisis were we not staring famine in the face’ (No. 78). One problem was the impossible situation of ‘working in an organisation that fosters “sustainable development” but is tightly controlled by several repressive governments’ (No. 60). State control over data and information led to personal disillusionment with the humanitarian sector, as shown in this scornful example:

*The government in my host country has received a lot of international support, partially because of its success in the healthcare arena. Yet, I personally witnessed several instances of manipulating health statistics, from maternal and infant deaths going unreported in my own hospital (so as not to hurt the country's mortality rates data) right up to the health ministry bragging to international papers that their new national health insurance scheme covered almost twice as many people as it actually did* (No. 47).

Donors were mentioned in the context of power relations: they had the ultimate say, but their procedures and requirements were perceived as counterproductive, hampering effective aid delivery. Below is one example of such a narrative:

*These tales highlight the biggest problem that the humanitarian community faces in aid delivery: donors. They all apply their own rules to how money can be spent, according to their own priorities. Rules are drawn up by bureaucrats in Washington, Brussels, London and elsewhere, with limited consideration to the needs on the ground. Donors apply disparate rules on procurement procedures so each purchase we make often must be done differently. Donors only care how their money is spent. They care little of the overall effectiveness or efficiency of the organisation spending the money. If we get it wrong, we must reimburse the donor's funds. The result is significant unnecessary work and delays. The problem, in short, is no effective coordination between donors* (No. 88).

The lack of donor coordination compounded the lack of autonomy, competence, and effectiveness among organisations. One author stated: ‘Through no fault of its own, the NGO sector has no autonomy. It is always beholden to its donors. And so is the UN’ (No. 68). Another confessed to a breakdown of purpose: ‘We used to call it “going to the dark side”. After years of struggling valiantly with local corruption, no resources and always praying to the almighty “donor Gods”, every NGO worker has had the thought: “Working for the United Nations would be so much easier”’ (No. 68). One article stated the perceived issue of system dysfunction with great clarity:

*With more than 10 years' experience in the humanitarian sector, working in logistics and supply chain, I have faced many challenges. The most frustrating by far? Seemingly arbitrary and nonsensical donor requirements … [I]t is vital that we openly discuss the dysfunctional outcomes resulting from donor requirements that are too strict* (No. 88).

These narratives are frankly critical of power dynamics in the humanitarian sector. The adage that ‘money is power’ was on full display; structural power flowed from donors through NGOs. Much criticism was reserved for the ‘mostly old, mostly white, mostly men from privileged backgrounds’ (No. 56). Notably, attention was directed at international aid workers, with little recognition of the contributions of national and local humanitarian staff. Criticism is harsh of an industry which employs a ‘motley crew’ of people ‘who no longer seem to care about the people they work with and for’, being ‘beholden to … donors’ and the power of bureaucrats ruling from headquarters. The narratives denounce management, poor support structures, and ill‐treatment, pouring scorn on ethically dubious practices related to organisational funding and promotion.

Host countries did not escape blame either. That nation‐states resorted to bureaucracy, obstruction, and obfuscation to restrict humanitarian space and action was a significant source of frustration. The narratives allowed for sharing personal reflections on issues within the humanitarian system, and the challenges therein, as well as on the part that different social actors played in making this a broken system. Essentially, they reflected a strong sense of frustration with power relationships within the humanitarian system.

### 3.3 Systemic issues within the humanitarian sector

Our third theme captures systemic issues within the sector, regarding: (i) limited career‐building opportunities; (ii) ineffectiveness and inefficiency in ways of working; (iii) safety, sexual misconduct, and duty of care; and (iv) racism and colonial continuities.

Concerns with career‐building challenges—entering the aid sector, promotion within, and exit strategy—loomed large. Women vis‐à‐vis men, as well as national staff vis‐à‐vis international staff, mentioned notable difficulties in this regard. One clearly articulated issue was the privilege enjoyed by international staff over their national colleagues, with respect to promotion and holding positions of decision‐making, as described in these excerpts:

*I started my career as a humanitarian worker in Sudan. A year out of university, after a brief interlude teaching English, I volunteered for an aid agency. Despite being no more committed or able than my Sudanese colleagues, I found myself rising rapidly through the ranks. After a few months, I was put in charge of a team of sanitation engineers, and within a year I was logistics manager leapfrogging a dozen national staff and in charge of a £1m budget* (No. 15).

*Despite having worked in this industry for so long I feel little has changed. Decisions about assignments are still very much based on who you know; how well you kiss up to someone and how well you ‘market’ yourself* (No. 37).

Both young, talented workers, and older, experienced workers, felt that their skills and abilities often went unrecognised. Such frustration is captured in these two examples:

*In the buildup to the summit [WHS], I hoped to see the current leadership empower young and alternative leaders‐in‐waiting, but instead I feel it's grown more defensive, micromanaging humanitarian operations and actively preventing the growth of emerging talent, especially anyone who could challenge them* (No. 52).

*We all know that breaking into this field is not easy. It requires time, personal and financial sacrifices, great connections and a good dose of humility. But I had underestimated how big an obstacle ageism would be* (No. 60).

Strong disillusionment was expressed regarding the ways in which power and privilege were wielded by fellow aid workers who chose to block access to other people's promotion. In the words of one aid worker:

*At the height of my cynicism about sexism in the workplace, I often made it a secret hobby to observe the power dynamics in a meeting. It wasn't just how men behaved towards women in management positions that baffled me, but how some women took other women less seriously too. Some of the more senior women participate, either consciously or unconsciously, in perpetuating the status quo and in coming down harshly on other female colleagues that are still making their way in the organisation and hoping to climb up the ladder. It is as if a woman has to pass suffer and then survive the same discriminatory practices to prove that she is worthy to remain and to move ahead* (No. 37).

At the core of many critical reproaches were portions of text highlighting the ineffectiveness and inefficiency of the humanitarian sector. Authors offer scenarios detailing unsustainable, outdated, and malfunctioning intervention approaches, lengthy and unproductive coordination processes across humanitarian stakeholders, and transient workforces made up of underperforming staff. As shown in the following excerpts, they blamed aid organisations for focusing on coverage rather than impact on beneficiaries, and prioritising organisational efficiency rather than effectiveness—issues exacerbated by high staff turnover:

*Often whatever was implemented by the previous expert is questioned by the next one, and so the process starts over again. As a consequence, we're underperforming significantly, and our combined efforts barely touch the tip of the iceberg* (No. 78).

*In the eyes of senior management, a successful humanitarian operation is based on two key indicators: how much money you raise with the donors and how many beneficiaries you have reached with the aid money you have been given. However, in my experience, what is not measured is how well you have managed projects in addressing the real needs of the intended recipients, how accountable you have been to them, and how quickly you have been able to address their urgent needs in humanitarian emergencies* (No. 95).

*Thus, it is crucial that the humanitarian sector here has access to people who can build and lead a crisis response, who continuously push for action, and who think practically and fast. Yet this crisis has an incredibly high staff turnover. And in some cases, organisations are simply unable to fill positions permanently. This means we end up reliant on surge staff and rotating experts. These people are flown in from head office to support a project, but in practice spend their time conducting a seemingly endless amount of assessments and meetings. As quickly as they arrive, they are gone – and another person comes in, and the cycle starts all over again* (No. 78).

Safety and sexual misconduct were issues that prompted authors not only to share their experiences of danger, but also to call explicitly for institutional change. As one author explained: ‘Often we work in insecure environments, where the rule of law means little and the roads can turn into complete quagmires during rainy season: security guards and 4×4s are essential to enable us to do our jobs effectively and safely’ (No. 36). Some expressed a clear‐eyed view of the inherent insecurity of humanitarian work, and awareness of how levels of insecurity differed for humanitarians vis‐à‐vis local civilians. For instance:

*My job has taken me to many countries and as you'd expect, I have found myself in a handful of dangerous situations where I looked death in the eye. None of these incidents deterred me continuing my work though. Instead each one convinced me that this is part and parcel of the work, and reminded me that the insecurity we face ourselves sometimes is often a fraction of what civilians in conflict situations find themselves in every night and day* (No. 37)

Some perceived real fear given perceived vulnerabilities owing to gender or nationality. For example:

*As a half‐Israeli half‐British woman … I have had to deal with speculation about my background and motives day in, day out while on mission in Muslim countries … It grates on you pretty quickly, and soon it becomes easier just to keep things vague, particularly when working in a place where your nationality may make you a target … At the local field office level, I always feared I could be made an example of in a cruel and sadistic way* (No. 41).

Another author recounted an example of sexual misconduct, with no pathway to recourse:

*I was working with a small NGO in an emergency situation, living and working 24/7 in a compound, just the six of us – three other women, two men. One day, cooking in the kitchen, one of the guys pulls down my T‐shirt to reveal my breasts. I was so shocked I didn't know what to say. So I made a joke, saying this was something I should report to HR. He was HR* (No. 7).

Many authors were speaking out because work environments were ‘untenable’ and needed urgent reform, prompting radical soul‐searching. Consequently, one wrote:

*In the same way that we need the humanitarian leadership and international community to engage on issues of violence against civilians, we also need those same leaders to engage on issues of violence against humanitarian staff. The current environment is untenable. Humanitarians cannot be effective at their jobs when the risk of violence against them or their friends is not just a possibility, but a probability. We have done everything we can. But now, we need help* (No. 26).

This called for reflections on an institution's duty of care to its staff, specifically with respect to its systems of support and due process. As the following authors explained:

*I didn't report the rape to the police because I had to leave the country straight away. After I finished the consultancy, I reported it to the agency I was working for. (The delay was because I wanted my work to be judged on its merits alone.) In response, the agency revised its written advice for employees and visiting consultants. Now it warns them about the dangers of spiked drinks and, if the worst should happen, the importance of getting an immediate HIV‐prevention kit. My contact, a woman, spent a lot of time talking to me. This meant the world to me* (No. 30).

*In the end, finding a workable solution came from the support network around me. My employers stood by me every step of the way, offering the possibility of flexible working conditions to respond to my family's needs* (No. 43).

Lastly, one issue of grave concern centred on systemic racism and colonial continuities. While some authors had reproduced prejudices and stereotypes pertaining to local staff ‘versatility’ or community ‘ignorance’, others reflected explicitly on their (white) privilege, as shown below:

*The rationale for my promotion was that, as a non‐national, I would be immune from participating in corruption that national staff may be vulnerable to; an unfortunate assumption of assuming the worst of national staff. So, having originally set off with ideals about creating global equality, I found myself in a scene often depicted in sepia colonial photos – white people in management seated at the front and Africans around the edges in junior roles* (No. 15).

*When one lives surrounded by well‐intentioned, yet gravely mistaken colonialists, missionaries and aid workers, you settle into a jaded state fairly quickly. I live in a town full of westerners who don't integrate. Fraternising with the natives is next to unheard of – unless, of course, you are sleeping with them. There are western restaurants, and local ones. The signs outside don't say ‘no blacks allowed’ but the prices do* (No. 48).

*Then there's the fact that so often we do jobs in Africa that we would never be allowed to do in our home countries. Simply because we are white, we are western, and we know what is best. ‘TIA’ (This is Africa) has become an excuse to do whatever we damn well please* (No. 48).

These reflections, however, also engendered a subtle caricature of aid workers as caught in systems of harm, where ‘speaking out could cost them their job’, and the profession of humanitarian work as ‘little aristocracy’. It seemed hard, for them, to escape their own caricatures. In their words:

*We know how large Africa is, yet we know that our admirers in our home countries don't. We spoon feed them the story of a broken, yet beautiful, continent (or country depending on your cultural intelligence) knowing they will hang on our every ostentatious word. While this is racism, in this form it is harder to detect. There are no sit‐ins, protests or rallies. We have made it seductive, sexy even, through victimising those we are helping. Instead, our white guilt has fashioned this new face of racism, equally as dangerous and scarily subtle. Often the beneficiaries of our aid and mission sit idly by because they know speaking out could cost them their job, their support, their stability. To brush aside this as an issue is the epitome of what racism is* (No. 48).

*Awareness of your own privilege in relation to the rest of the world is often a motivator for people drawn to humanitarian or development work. The ironic twist is that in this profession we are transformed from unremarkable young people into a little aristocracy* (No. 35).

*It is not about eliminating privilege. It is the recognition of our privilege that motivates us to go to places like South Sudan or the Democratic Republic of the Congo. It is our privilege that makes us put our hands in our pockets to help those less fortunate than ourselves* (No. 36).

These narratives illustrate vividly how institutional power operates. Power differentials between staff based on nationality, gender, and race resulted in discrimination, exclusion, and at times, criminal behaviour. Misguided notions of humanitarian action held the ‘international’ humanitarian as the one with superior knowledge and skills, resulting in systematic exclusion of ‘national’ or locally‐hired humanitarians from positions of influence. Some aid workers condemned this position, as an ill‐informed caricature, vehemently questioning the popular perception of humanitarians as ‘heroes’. They pointed to the ‘ironic twist’ of this profession (No. 35) in transforming power and privilege: namely, humanitarian actors, motivated to serve humanity because of suffering and injustice, actually wielded power in neocolonial and exclusionary ways. While the articulation of such concerns is not new, what came through was a call for reforms to create workplaces that are safe and respectful of differences and diversity.

## 4 DISCUSSION

Our study captured public discourse data, written by aid workers between 2015 and 2018, and revealed its value as a historical archive of contested narratives of humanitarian work. The SAW series itself is a sign of changing times. Aid workers did not see themselves as volunteers with a ‘selfless soul’ (Houldey, 2017, p. 1) but as professional workers with explicit career aspirations. Their narratives revealed widespread discontent about humanitarian life—but they also planted the seeds of change with regard to addressing issues they collectively raised, such as a lack of institutional accountability, coloniality, racism, and sexism. The series is of particular interest in proffering personal insights into how aid workers have characterised the power and privilege wielded by their colleagues, organisations, and donors. It allows us to reflect on where, in terms of public discourse over the past five to eight years, change has come. This is useful, not only for reflecting on meaningful facets of humanitarian culture, as told by aid workers themselves, but also for identifying turns in public discourse and calls for reform within the humanitarian system.

### 4.1 SAW narratives as powerful discourses of discontent

Articles in the SAW series likely echoed the opinions of a small group of internationally mobile staff. Their narratives may not be seen as an account of all of the ills that faced humanitarian actors, rather the tip of a very large iceberg. The versions of humanitarian life presented in this corpus of archival data are nonetheless significant. As James (2022, p. 477) argued in her analysis of humanitarian fables, ‘stories are rich repositories of meaning’. For instance, stories can consolidate consensus on ways of working—‘consensus about why things are the way they are in an organization’ (James, 2022, p. 465). The fables recounted among MSF international staff in the eastern Democratic Republic of the Congo served as ‘discourses of power’ (James, 2022, p. 479) to rationalise institutional practices, in the form of warnings about breaches of security (through risky sex with locals), trust (given ambiguous loyalties), and corruption (through vulnerability to scams). In so doing, they served to consolidate the distancing of international staff from local citizens and even national staff, reinforcing through symbolic power the ‘micro‐politics of inequality’ (James, 2022, p. 478) in humanitarianism.

The stories embedded in the SAW series also have a form of symbolic power. Like fables, they are published anonymously and conveyed as storytelling to a largely lay audience. In this case, though, they convey personal and collective warnings about how oppressive aid structures can block career opportunities and about how oppressive management will bake power differentials into gender and racial injustices. The stories, implicitly or explicitly, also convey the warning that staff disillusionment and rapid turnover will worsen the inherent inefficiencies of present‐day forms of the humanitarian sector. They function as parting shots by an often‐disenchanted workforce, reflecting on a mismatch between expectations and experiences. The following excerpt is a striking example of an author conveying the purpose of ‘stories’, as ‘tales’ that convey dark ‘secrets’ about social representation and actual reality: ‘These tales highlight the biggest problem that the humanitarian community faces in aid delivery: donors’ (No. 88).

Of course, the SAW narratives capture much of what has previously been written in the academic and grey literature about humanitarian work environments, daily life, and the absurdity of situations experienced on the frontlines of humanitarian assistance (see, for example, Bergman, 2003; Cain, Postlewait, and Thomas, 2004; Alexander, 2013; Roth, 2015). Likewise, they echo the concerns raised by autobiographical and ethnographic works, with respect to the ineffectiveness and inefficiency of the humanitarian system, and the multitude of personal challenges experienced by aid workers (see, for example, Cain, Postlewait, and Thomas, 2004; Kleinman, 2007; Orbinski, 2008; Allen, 2015; Malkki, 2015). Yet, they push critical discontent into new forms of communication, via the platform of mainstream media. It is for this reason that they are important: these discourses are public because they are performative, wanting to sow change in public imagery about who humanitarians really are, and what issues plague the humanitarian system.

Public discourse is a strong form of social communication and social representation, through which discontent and criticism can help to usher in social change (Ongenaert and Joye, 2019). It beholds us then to ask: what has changed with respect to an aid worker's experiences of everyday humanitarian life since the 2015–18 period? At the beginning of our analysis, we asked two questions: (i) how did authors frame their work and appraise humanitarian structures?; and (ii) how did this public discourse reflect and amplify contentious humanitarian issues of the time? We now discuss how such issues have engendered calls for specific reforms in the present day. We frame this reflection broadly in terms of shifting power in humanitarian systems, and specifically discuss donor power, coloniality and racism, sexual abuse, and duty of care.

### 4.2 Shifting power in humanitarian systems

Power continues to be affirmed as a critical issue for generating reform of the humanitarian system. Current criticism is strongly worded: ‘Western humanitarian aid is frequently imperial in style, colonial in its practices and racist in assumptions of its operational superiority’ (Slim, 2022, p. 236). Power operates overtly and covertly, largely structurally, concentrating the ability to shape the agenda in the hands of wealthy countries and individuals. Power flows through organisations that are funded, often by former colonial powers, and attitudes that reek of paternalism and privilege. For example, international donors are typically governments of liberal democratic countries and members of the Organisation of Economic Co‐operation and Development (Slim, 2022). Power, however, is not unidirectional, with receiving states and civil society interacting, and at times asserting themselves, in a complex adaptive power system (Moon, 2019).

Scholars and practitioners have been critical of *donor power* and the ensuing positioning of humanitarian initiative (Owen, 2021). This spawns fraught interactions between aid workers, organisations, and donors. Indeed, some of the SAW articles point to the plural hierarchies of power and privilege within humanitarianism by referring to ‘almighty “donor Gods”’ (No. 68), creating institutional dependencies, with adverse impacts on staff motivation and outputs. By contrast, new forms of humanitarian action ask us to build sustainable, resilient systems ‘with people’, not ‘for people’ (Slim, 2020b), to put in place ways of working that are neither unintentionally nor explicitly top‐down structural articulations of power and privilege. Aid workers writing for the series viewed the typically rigid requirements imposed by donor contracts as the root of many personal, operational, and logistical challenges, among them delays in aid delivery, a lack of coordination, and neocolonial practices. These are known complaints, ones which have generated a rethink of the humanitarian sector and a push to change its hierarchical structure, towards more distributive networks of information and flows of resources (Bennett, 2018; Currion, 2018). And yet, while donors have been quick to endorse localisation, progress has been excruciatingly slow, as they remain reluctant to surrender power (Ainsworth, 2024).

This brings us to humanitarian discourses on *coloniality and racism*. The discourses of decolonisation and anti‐racism are distinct, but closely related and overlap with the localisation discourse (Robillard, Atim, and Maxwell, 2021). Within the SAW series, few authors explicitly discussed racism in the humanitarian sector; indeed, the narratives often reproduced mainstream prejudices and stereotypes (about ‘helpless’ beneficiaries) and an inflated sense of their ability to bring about change (with undertones of white saviourism). Yet, they spoke of power and privilege, and would confront racism, when speaking about representations of the continent of Africa:

*We spoon feed them the story of a broken, yet beautiful, continent … While this is racism, in this form it is harder to detect. There are no sit‐ins, protests or rallies. We have made it seductive, sexy even, through victimising those we are helping. Instead, our white guilt has fashioned this new face of racism, equally as dangerous and scarily subtle* (No. 48).

Since 2020, the year marked by the deaths of George Floyd and others in the US and the re‐emergence of the BLM movement, the calls for decolonisation and anti‐racism have loomed large in humanitarian public fora: we now see ‘decolonising aid’ and ‘addressing racism’ discussed in blogs, interviews, webinars, and clips produced by humanitarian stakeholders and published across social media platforms and humanitarian outlets such as the CHA, Devex, the Humanitarian Practice Network, openDemocracy, and The New Humanitarian (Cornish, 2019; Ali and Romain Murphy, 2020; Aloudat, 2021; Khan, 2021; Khan, Dickson, and Sondarjee, 2023). Indeed, as argued by Dunivin et al. (2022) in their analysis of large‐scale news and social media data focused on BLM, what is captured by the media can instrumentally shift public discourse on systemic racism; certainly, mainstream and social media can dramatically amplify protests, social change, and attentiveness to social movements.

These current humanitarian discourses centre inter alia on the questions about who gets to decolonise aid (Aloudat, 2021; Khan, 2021) and how to address the structural barriers deeply enshrined in the architecture of the humanitarian system (Ali and Romain Murphy, 2020). Some scholars and practitioners are now challenging the idea that ‘decolonising aid’ and structural change can be achieved through incremental reform of existing practices, rather than through a systematic overhaul of the system, stripping hierarchies of power (James, 2022). Furthermore, organisations have been confronted with intensified demands from members of their workforce, the public, and academic scholars to investigate and address colonial continuities and racism within the ranks (see, for example, McVeigh, 2020; Liesner et al., 2020; Slim, 2020a; Bian, 2022; Khan et al., 2021; Strohmeier et al., 2024). These have included calls to decolonise aid narratives and imagery, that is, to rethink critically terminology that creates an ‘us and them’ dichotomy like ‘Global North’ and ‘Global South’, and ‘charity advertising, which often depicts black children as distressed or severely malnourished’ (Elahee, 2021, p. 8), as well as to audit ‘all hiring processes (including recruitment channels) at all levels of the organisation, with identification and exploration of barriers hindering the recruitment and retention of a diverse workforce’ (Liesner et al., 2020).

What many aid organisations have done is to respond to calls for antiracism and decolonising social movements by implementing surveys, awareness‐raising campaigns, and newly‐developed policies and guidelines (see, for example, Task Force on Addressing Racism and Promoting Dignity for All in the United Nations Secretariat, 2021; MSF Eastern Africa, 2022; Oxfam GB, 2023; Strohmeier, Musizvingoza, and Sajnani, 2024). Little is known about the efficacy of such initiatives, and some doubt their sufficiency (see, for example, The New Humanitarian, 2021; Prom‐Jackson, 2023).

Greater attention to systemic racism is thus emerging, but as with decolonisation, it is ‘unfinished business’ (Currion, 2020, p. 2). Writing for The New Humanitarian in 2020, aid worker and humanitarian consultant Paul Currion (2020, p. 2) confessed that: ‘Two years ago, I wrote a paper for the Overseas Development Institute, Network Humanitarianism, about the systemic challenges facing humanitarian organisations, the underlying origins of those challenges, and possible solutions … I didn't use the word “racism” once in that paper’. In brief, it is now noted that many studies on aid and the lives of humanitarian professionals ‘reflect on the hierarchical divide along the lines of class exclusivity and foreign status prestige, although they not always accommodate the salience of race’ (Bian, 2022, p. 3).

Humanitarian workers are susceptible to power and abuse in various ways and forms. *Sexual abuse and harassment* became a prominent issue in humanitarian public discourse after the misconduct of Oxfam staff in the aftermath of the 2010 earthquake in Haiti was made public, requiring vigorous institutional responses. Discussions on safeguarding and sexual discontent have dominated the humanitarian agenda over the past decade (Sandvik, 2019), while the #AidToo movement leveraged a digital discourse in 2018 about assault and sexual harassment within the aid industry (The New Humanitarian, 2019), forcing aid organisations to ‘look inward’ at their own governance and accountability mechanisms. Many had internal processes to report forms of sexual abuse, but there was a lack of awareness of and especially confidence in internal processes, as well as a lack of sensitivity, capacity, and of will to enforce institutional policy. MSF, for instance, had to revisit its policies and strengthen internal processes; it now reports on safeguarding annually (MSF, 2023). Many organisations have since rushed to adopt zero‐tolerance safeguarding policies on any form of abuse on the basis of gender, and public discourse on sexual abuse and harassment within organisations has decreased in humanitarian media in recent years. And yet, much work remains to be done to tackle this issue and to establish organisational work environments in which humanitarian staff feel safe within their own sector (Cornish, 2021).

It is incumbent then on organisations to minimise harm and shoulder responsibilities diligently. For humanitarian organisations, *duty of care* largely refers to their responsibility and obligation to minimise risk of harm and to maximise the well‐being of staff and the communities they assist. In Steven Patrick Dennis versus the NRC, the courts ruled that a failure to take this duty seriously has legal and financial implications (Hoppe and Williamson, 2016). This case and experiences of the 2014–16 Ebola outbreak highlight the importance of aftercare following any illness or injury that may have resulted from exposure to harm (McDiarmid and Crestani, 2020). In addition, new literature has highlighted the importance of staff concerns, calling for humanitarian organisations to expand and improve mental health services and psychosocial support for their employees (Strohmeier, Scholte, and Ager, 2019). Faced with potential and legal liabilities, many organisations have meanwhile enacted duty of care policies. For instance, concerns about the mental health of humanitarian aid workers, as opposed to the mental health burden of people living in crisis settings, have now been legitimised (see, for example, Strohmeier, Scholte, and Ager, 2018; Young and Pakenham, 2021; Parvin et al., 2022). More recently, as a sign that the humanitarian system is taking this obligation seriously, the Inter‐Agency Standing Committee, the humanitarian coordination platform of the UN system, issued the *Minimum Standards on Duty of Care in the Context of COVID‐19* (IASC, 2020). While compromised mental health and well‐being remains an issue within the occupational group of humanitarian workers, public discourse on this topic has waned.

## 5 CONCLUSION

The main strength of this study is that it highlights gaps in knowledge: the discourse on humanitarianism has seldom been authored by aid workers themselves, and little is known about how their public discourse can influence issues over time. Arguably, the SAW series—representing a historical archive of humanitarian life—was authored by aid workers who wanted to plant seeds of change with respect to social representations within their sector. They did this through caricature, soul‐searching, and public communication. We are mindful that Dunivin et al. (2022, p. 1), in their study, were able to demonstrate that BLM media postings succeeded in ‘drawing attention to antiracist theory in a large‐scale and consistent way’. In the humanitarian sector, by contrast, it is hard to demonstrate that public discourse has led to concrete changes—because many such changes are still un‐systemic. We point to several other study limitations, too. First, the SAW narratives were selected and edited by *The Guardian* based on unknown criteria (the newspaper did not respond to our request to share this information); however, its website headlines suggest that selection was made on the basis of issues discussed in multiple submissions. Second, the authors wrote anonymously for publication. This prevented us from comparing sociodemographic authorship data, although most articles seem to have been written by international staff, a small fraction of humanitarian stakeholders.

In conclusion, our analysis is predicated on the view that public discourse data sustain powerful discussions about social and systemic change. The SAW series represents a historical collection of humanitarian life. Since it is public, albeit anonymous, its discourse of discontent has symbolic power, calling for greater accountability, equity, and justice in remaking the future of the humanitarian sector.

## ETHICS STATEMENT

This paper reports analysis of secondary sources.

## Supporting information


**Table S1** ‘Secret Aid Worker’ articles included in the thematic analysis.

## Data Availability

The data that support the findings of this study are openly available in the Secret Aid Worker repository at https://www.theguardian.com/profile/secret-aid-worker.

## ENDNOTE

^1^
All supplementary materials can be found online in the Supporting Information section at the end of the article.
