# Supplementary material for: Public discourse narratives: from ‘Secret Aid Worker’ discontent to shifting power in humanitarian systems
Source: Disasters. 2024 Jul 15;49(1):e12651. doi: 10.1111/disa.12651 (PMC11603526; doi:10.1111/disa.12651)
Supplement: Supplementary file 1 — Table S1 ‘Secret Aid Worker’ articles included in the thematic analysis. [file DISA-49-e12651-s001.docx]

| **Table S1.** ‘Secret Aid Worker’ articles included in the thematic analysis | | |
| --- | --- | --- |
| **No.** | **Print date** | **Title of articles published anonymously in *The Guardian*** |
| 1 | 2015, April | Is there such a thing as being too French? |
| 2 | 2015, April | After years in the field, I worry I've lost my compassion |
| 3 | 2015, June | Flaky aid boys and other development love-life disasters |
| 4 | 2015, June | It's one standard for local staff and another for expats |
| 5 | 2015, June | 'Has anyone worked out if celebrities are worth the effort?' |
| 6 | 2015, July | I'm a sanitation specialist but I'm squeamish about poo |
| 7 | 2015, July^1^ | Groping in close quarters |
| 8 | 2015, July^1^ | Isolation in the workplace |
| 9 | 2015, July^1^ | Systematic harassment of local staff |
| 10 | 2015, July^1^ | Undiplomatic behaviour |
| 11 | 2015, July^1^ | Extra-marital affairs with disaster victims |
| 12 | 2015, July^1^ | Offensive Skype messaging |
| 13 | 2015, July^1^ | Pornography in meetings |
| 14 | 2015, August | Development work broke a piece of me |
| 15 | 2015, August | There is still racism within humanitarian work |
| 16 | 2015, September | Sometimes I go to supermarkets to escape the loneliness |
| 17 | 2015, September | We need to listen to humans, not follow programmes |
| 18 | 2015, September^2^ | I was told I was “clearly not committed” to the work |
| 19 | 2015, September^2^ | Never work for an organisation without proper security measures |
| 20 | 2015, September^2^ | My body gave up. It simply went on strike |
| 21 | 2015, September^2^ | My stomach churned at the thought of taking on another assignment |
| 22 | 2015, September^2^ | I walked into my office and physically collapsed |
| 23 | 2015, September^2^ | I feel guilty about saying I need a long break |
| 24 | 2015, September | As a woman, I'm seen as a piece of meat |
| 25 | 2015, October | 10 of the village girls have been sold |
| 26 | 2015, October | There is a new trend of sexual violence in South Sudan |
| 27 | 2015, October | Is there life beyond the field? |
| 28 | 2015, November | I’m a peacekeeper, but I still like humanitarians |
| 29 | 2015, November | Two years after Haiyan, the aid economy is flourishing |
| 30 | 2015, November | The journey to trace my childhood ended in rape |
| 31 | 2015, November | NGOs rarely say no to corporate cash |
| 32 | 2015, December | Disposing of tampons in the jungle and other dilemmas |
| 33 | 2015, December | Secret aid worker: We are just cleaning up the mess politicians make |
| 34 | 2015, December | Working in a war zone I never thought I'd get trolled online |
| 35 | 2015, December | How can we fight inequality if we live as privileged expats? |
| 36 | 2016, January | It's unrealistic to expect us to live like monks |
| 37 | 2016, January | I feel disillusioned by the favouritism in my organisation |
| 38 | 2016, January | 'I was the obscure African girl in a room full of white faces’ |
| 39 | 2016, February | I hate calling refugees to tell them they're not getting resettled |
| 40 | 2016, February | Is humanitarian work a career for escapists? |
| 41 | 2016, March | Why I hide my Israeli identity |
| 42 | 2016, March | Buzzwords are killing development |
| 43 | 2016, March | My child has a life-limiting illness, was I right to go back to work? |
| 44 | 2016, March | No matter where we work, we are all part of The A-Team |
| 45 | 2016, March | Why do expats earn more than the rest of us? |
| 46 | 2016, April | Fixing the humanitarian and development divide |
| 47 | 2016, April | What should doctors do when we witness human rights abuses? |
| 48 | 2016, April | Who will save the white saviours from themselves? |
| 49 | 2016, May | Can only the childless and unattached manage the work we do? |
| 50 | 2016, May | I regret not speaking out for my LGBT colleagues |
| 51 | 2016, May | I'm sick of job rejections - is humanitarian work only for the elite? |
| 52 | 2016, May | 'It is time to pass the UN leadership baton to a new generation' |
| 53 | 2016, May | We were there to win hearts and minds, but every war gets its own comedy' |
| 54 | 2016, June | My bra stopped me from running into the path of gunfire |
| 55 | 2016, June | 'It's time to talk about the dark side of development comms' |
| 56 | 2016, June | 'High-level' really means a club of old white men |
| 57 | 2016, July | Consultants aren't the problem, it's the people who hire us |
| 58 | 2016, July | The field' is not a lab where you can experiment without consequence |
| 59 | 2016, July | When your dream job ends in depression |
| 60 | 2016, July | Is the humanitarian sector ageist? |
| 61 | 2016, August | The UK NGO sector is facing a funding crisis |
| 62 | 2016, August | How to find love, or at least a date, after the field |
| 63 | 2016, August | I want to help Afghanistan but I am an outsider in my own country |
| 64 | 2016, August | People are hungry and I have food that can't be delivered |
| 65 | 2016, August | I'd rather help people abroad than my own community |
| 66 | 2016, September | Some human rights lawyers are in it for vanity, not victims |
| 67 | 2016, September | Greece has exposed the aid community's failures |
| 68 | 2016, September | This is what happens when an NGO worker goes to the UN |
| 69 | 2016, October | It's ok to not love this job all the time |
| 70 | 2016, November | Sorry to disappoint you, but we can't all be Mother Theresa |
| 71 | 2016, November | Surely NGOs should embrace trade unions, not block them? |
| 72 | 2016, November | I'm waiting for deployment, caught between two lives |
| 73 | 2016, December | NGOs can be efficient, if it involves sacrificing staff |
| 74 | 2016, December | Happy Christmas from conflict zone number five |
| 75 | 2017, January | Does your employer have the right to know you're dating a colleague? |
| 76 | 2017, January | Development in Palestine is hostage to politics |
| 77 | 2017, February | Men have as many issues as women, we just don’t know what they are |
| 78 | 2017, March | When your crisis isn't cool enough to attract the right people |
| 79 | 2017, April | What I wish I could say to the people back home |
| 80 | 2017, April | We've lost our humanity to jargon and statistics |
| 81 | 2017, April | Volunteering in Greece made me see the west in a new light |
| 82 | 2017, May | Why don’t we practise what we preach about gender inequality? |
| 83 | 2017, May | Resettling refugees was the bane of our lives' |
| 84 | 2017, June | Charities have been gagged in the UK election – this is why |
| 85 | 2017, June | We don't take data protection of vulnerable people seriously |
| 86 | 2017, June | The problem with positive discrimination in NGOs |
| 87 | 2017, July | Mid-life crises hit earlier in the humanitarian world |
| 88 | 2017, July | We learned the hard way that all funding has strings attached |
| 89 | 2017, July | Why do we still value expats more than local staff? |
| 90 | 2017, August | The UK has lost its moral high ground partnering with the DUP |
| 91 | 2017, August | Do I have imposter syndrome or am I just not good enough? |
| 92 | 2017, September | Have smartphones replaced friendships out in the field? |
| 93 | 2017, November | Forced to have oral sex with a colleague: aid workers speak out on assault |
| 94 | 2018, March | No, abusive men in the aid sector won't 'grow out of it' |
| 95 | 2018, March | I saw aid for starving people spent on staff salaries through inept planning |

^1^ Seven short articles published at once under the heading ‘Sexual harassment and discrimination in the industry’.

^2^ Six short articles published at once under the heading ‘Your stories of mental health, PTSD and burnout’.
